# Supplementary material for: Within-host evolution of SARS-CoV-2 in an immunosuppressed COVID-19 patient as a source of immune escape variants
Source: Nat Commun. 2021 Nov 4;12:6405. doi: 10.1038/s41467-021-26602-3 (PMC8568958; doi:10.1038/s41467-021-26602-3)
Supplement: Supplementary file 1 — Supplementary Information [file 41467_2021_26602_MOESM1_ESM.pdf]

Supplementary Information

Within-host evolution of SARS-CoV-2 in an immunosuppressed COVID-19 patient as a source of immune escape variants

Sebastian Weigang, Jonas Fuchs, Gert Zimmer, Daniel Schnepf, Lisa Kern, Julius Beer, Hendrik Luxenburger, Jakob Ankerhold, Valeria Falcone, Janine Kemming, Maike Hofmann, Robert Thimme, Christoph Neumann-Haefelin, Svenja Ulferts, Robert Grosse, Daniel Hornuss, Yakup Tanriver, Siegbert Rieg, Dirk Wagner, Daniela Huzly, Martin Schwemmle, Marcus Panning

a

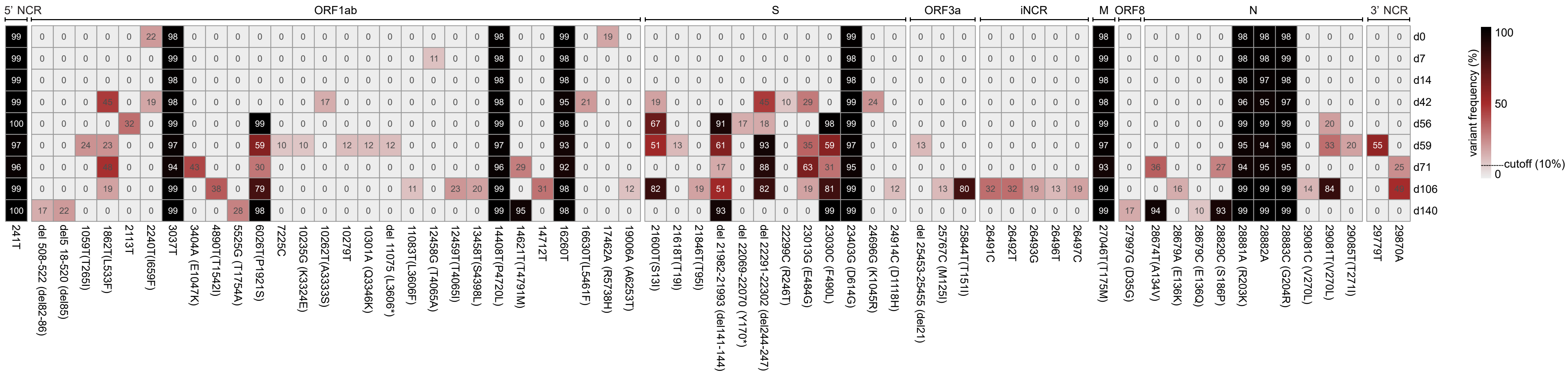

Supplementary Figure 1: SARS-CoV-2 whole genome sequence and analysis of viral genomes. Overview of the viral genome variations as shown in figure 2c from patient swab samples (day 0-140) in comparison to the Wuhan-Hu-1 reference sequence. The heatmap summarizes the positions in the viral genome and the variant frequencies in the different samples using a cut off value of 10%. The variant frequencies are indicated by the numbers in the boxes and by the color intensity at the right. The days of sampling are indicated at the right.

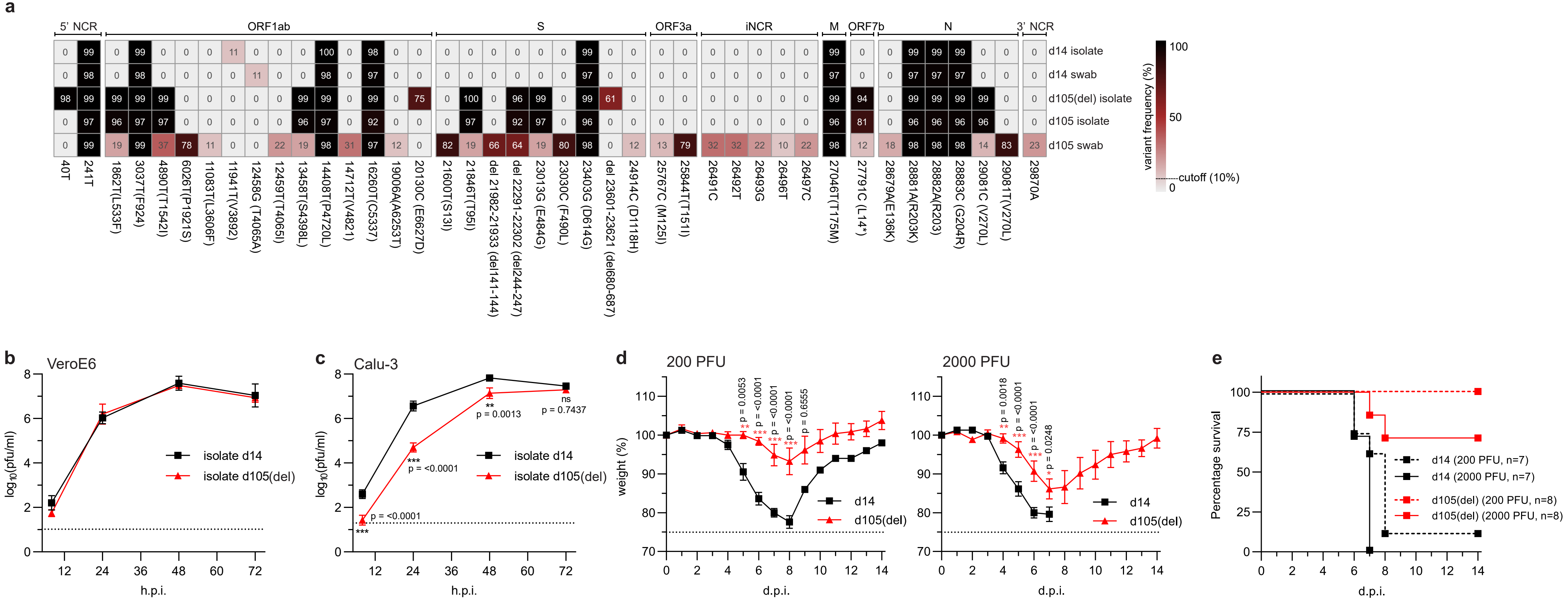

Supplementary Figure 2: Growth characteristics of the SARS-CoV-2 d105(del) variant. (**a**) Viral genome variations as shown in figure 4a from patient early (d14) or late (d105) swab samples and isolated viruses in comparison to the Wuhan-Hu-1 reference sequence. Variant d105(del) represents an early virus stock from VeroE6 cells with a 21 nucleotides deletion (del23601-23621) in 50% of the spike gene sequences, resulting in an eight amino acids SPRRARSV deletion. The heatmap summarizes the positions in the viral genome using a cut off values of 10%. The variant frequencies are indicated by the numbers in the boxes and by the color intensity at the right. (**b**) Vero E6 and (**c**) Calu-3 cells were infected with either of the two variants, d14 or d105(del), using 0.001 pfu/cell. At different time points post infection, cell culture supernatants were collected and viral titers were determined. The log-transformed titers are shown as means ± SD of results from three independent experiments. Significance was determined via two-way ANOVA with a Sidak´s multiple comparison test, **p<0.01, ***p<0.001, ns=non significant. The exact p-values are given in the figure. (**d** and **e**) *In vivo* infection experiments. Survival of 8 to 12 weeks-old K18-hACE2 mice intranasally infected with 200 or 2000 pfu of d14 (n=7, each) or d105(del) (n=8, each) viruses. The animals were euthanized if severe symptoms were observed or body weight loss exceeded 25 % of the initial weight. The weight loss is visualized as mean ± SEM. Significance was determined via two-way ANOVA with a Sidak´s multiple comparison test, *p<0.1, **p<0.01, ***p<0.001. The exact p values are visulized in the figure. Source data are provided as a Source Data file.

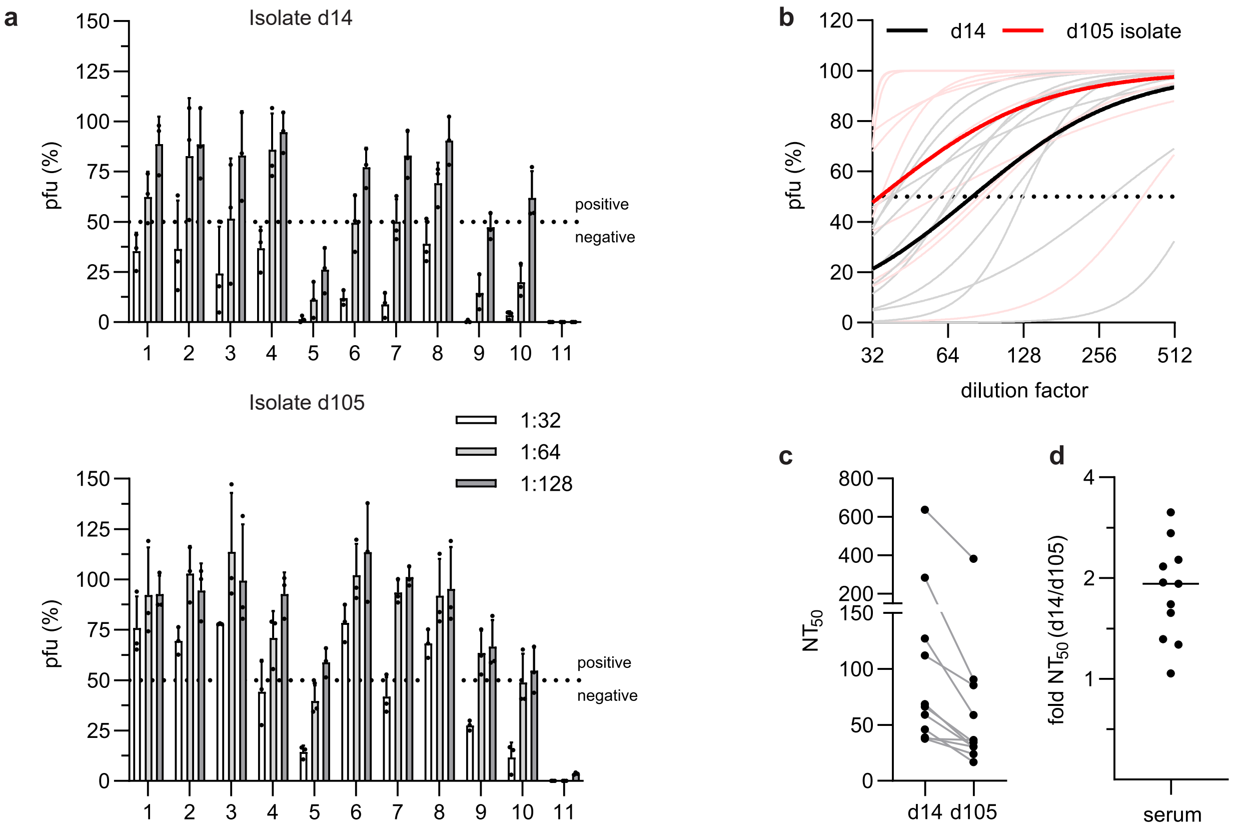

Supplementary Figure 3: Isolate d105 escapes neutralization by COVID-19 specific antisera like d105(del). (**a**) Neutralizing activity of immune sera against the patient's isolates. Sera 1 to 10 from convalescent COVID-19 patients and serum 11 from an individual after a SARS-CoV-2 infection and a following AstraZeneca-Vaxzevria vaccination. 100 pfu of d14 and Calu3-propagated d105 isolate without a deletion in the furin cleavage site were incubated for 60 min at room temperature with serial dilutions of the antisera. Virus neutralization was determined by plaque assay on VeroE6 cells. Virus titers are indicated as percentages (mean ±SD) of the titer of the untreated virus inoculum for the serum dilutions 1:32, 1:64 and 1:128. The dotted line indicates the cutoff value between positive (<50%) and negative (>50%) neutralization. Shown are the means of three biological replicates. (**b**) Curves for the calculation of the NT_{50} values via a non-linear regression of a broader range of antiserum dilutions (variable slope, four parameters). Shown are means (n=3) of three biological replicates. The results for each serum are presented in light red (d14) and light black (d105) and the mean values for all 11 sera are visualized in dark black and dark red for the d14 and d105 variants, respectively. (**c**) Visualization of the NT_{50} values determined from the data shown in panel b. The NT_{50} values for the two variants are connected for each serum. (**d**) The fold differences in NT_{50} between d14 and d105 were calculated for each individual antiserum using the values given in panel c. Source data are provided as a Source Data file.

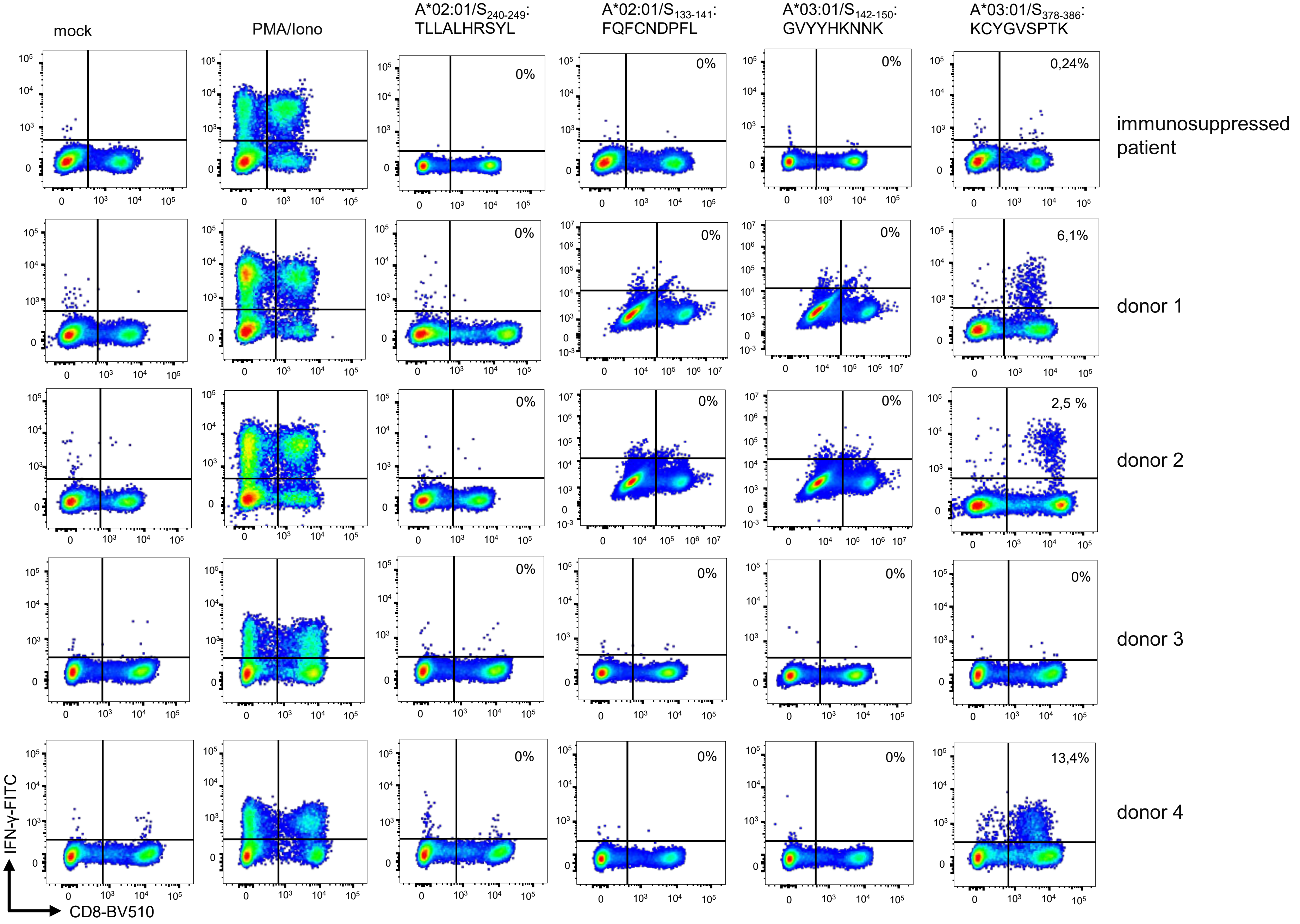

Supplementary Figure 4: Lack of detectable CD8+ T cell responses targeting the mutated regions in the spike protein. HLA-A*02:01/HLA-A*03:01-restricted CD8+ T cell epitopes overlapping with the del141-144 and the del244-247 deletions as well as a control peptide 378-386 in the S1 domain were predicted *in silico*. Peptide-specific CD8+ T cell responses were assessed by intra-cellular IFN production following a 14-day *in vitro* peptide-specific CD8+ T cell expansion. CD8+ T cells targeting the *in silico*-predicted SARS-CoV-2 S1-specific, HLA-A*02:01/HLA-A*03:01-restricted CD8+ T cell epitopes were neither detectable in the convalescent, immunosuppressed COVID-19 patient (upper panel) nor in four additional HLA-A*02:01/HLA-A*03:01 positive convalescent, immunocompetent COVID-19 patients (donor 1 to 4). CD8+ T cell responses targeting the immunodominant A*03:01/$S_{378-386}$ epitope are depicted as positive control, as well as mock-stimulated and PMA/ Ionomycin-stimulated cells.

Supplementary Table 1: Days according to the first positive qPCR test and respective clinical parameters.

Day	Ct	SARS-S1	SARSMIK-N	Virus isolation	NT (dilution factor)
-3		0.068	3.35		
-2		0.07	3.44		
0	25	0.07	3.61	negative	
6	16			positive	
8		0.073	3.26		
9		0.065	4.23		
10	19	0.075	10.9	positive	
12		0.119	27.9		
13		0.095	46.5		0
14	19			positive	
16	25	0.184	77	negative	
17		0.286	74.1		
19		0.332	85.5		
20		0.434	82.4		0
21	23			positive	
23		0.599	96.6		
24	18			positive	
25		0.561	98.1		
27	26			positive	
28		0.496	93.6		
29		0.685	102		
31	20			positive	
32		0.735	99.5		
34	17			positive	
37		0.659	89.4		
38	31			negative	
41		0.902	90.6		0
41		0.902	90.6		
42	27			negative	
43		0.782	115		0
44		0.846	117		
45	27	0.807	101	negative	
46	50				
49	27	0.878	107	negative	0
50		0.882	109		
52	28	0.841	113	negative	
53		0.986	125		
56	25	1.05	118	negative	
57		1.01	125		
58	28	0.83	117	negative	
60		0.878	111		
63	27	0.939	106	negative	
65	29	0.846	113	negative	
66		1.01	123		
67		0.889	138		0
71	30	0.966	120	negative	
72		0.984	113		0
105	23			positive	
106				negative	
113	34			negative	
115	29			negative	
119	31			negative	
122	31				
123		0.872	109		0
126	36				
140	26			negative	
141		5.61	108		64
143	29	5.68			64
145	34				
146	39				
147		6.13	95.6		64
149	45				
150	45				64
154	34			negative	
161	45	3.4	104		64
167	45				
174	45				
175		5.84	84.2		64
184					64
189	45				

Supplementary Table 2: List of GISAID accession numbers of sequences from Freiburg.

GISAID Accession	Metadata	GISAID Accession	Metadata	GISAID Accession	Metadata
EPI_ISL_852733	Freiburg area	EPI_ISL_852720	Medical center Freiburg	EPI_ISL_852708	Freiburg area
EPI_ISL_852729	Freiburg area	EPI_ISL_852719	Medical center Freiburg	EPI_ISL_852707	Medical center Freiburg
EPI_ISL_852727	Medical center Freiburg	EPI_ISL_852718	Medical center Freiburg	EPI_ISL_852706	Freiburg area
EPI_ISL_852693	Freiburg area	EPI_ISL_852717	Freiburg area	EPI_ISL_852705	Freiburg area
EPI_ISL_852772	Medical center Freiburg	EPI_ISL_852771	Freiburg area	EPI_ISL_852704	Freiburg area
EPI_ISL_852784	Medical center Freiburg	EPI_ISL_852716	Medical center Freiburg	EPI_ISL_852703	Freiburg area
EPI_ISL_852781	Medical center Freiburg	EPI_ISL_852715	Medical center Freiburg	EPI_ISL_852702	Freiburg area
EPI_ISL_852778	Medical center Freiburg	EPI_ISL_852714	Freiburg area	EPI_ISL_852700	Freiburg area
EPI_ISL_852775	Medical center Freiburg	EPI_ISL_852713	Medical center Freiburg	EPI_ISL_852699	Freiburg area
EPI_ISL_852701	Medical center Freiburg	EPI_ISL_852712	Medical center Freiburg	EPI_ISL_852698	Medical center Freiburg
EPI_ISL_852765	Medical center Freiburg	EPI_ISL_852711	Medical center Freiburg	EPI_ISL_852697	Freiburg area
EPI_ISL_852792	Medical center Freiburg	EPI_ISL_852748	Medical center Freiburg	EPI_ISL_852696	Freiburg area
EPI_ISL_852773	Medical center Freiburg	EPI_ISL_852802	Medical center Freiburg	EPI_ISL_852695	Medical center Freiburg
EPI_ISL_852744	Freiburg area	EPI_ISL_852804	Freiburg area	EPI_ISL_852694	Freiburg area
EPI_ISL_852743	Freiburg area	EPI_ISL_852737	Freiburg area	EPI_ISL_852692	Freiburg area
EPI_ISL_852741	Medical center Freiburg	EPI_ISL_852801	Medical center Freiburg	EPI_ISL_852691	Medical center Freiburg
EPI_ISL_852738	Freiburg area	EPI_ISL_852785	Medical center Freiburg	EPI_ISL_852690	Medical center Freiburg
EPI_ISL_852731	Freiburg area	EPI_ISL_852786	Medical center Freiburg	EPI_ISL_852769	Medical center Freiburg
EPI_ISL_852723	Freiburg area	EPI_ISL_852783	Medical center Freiburg	EPI_ISL_852689	Freiburg area
EPI_ISL_852710	Medical center Freiburg	EPI_ISL_852800	Medical center Freiburg	EPI_ISL_852688	Medical center Freiburg
EPI_ISL_852811	Freiburg area	EPI_ISL_852799	Medical center Freiburg	EPI_ISL_852687	Freiburg area
EPI_ISL_852732	Medical center Freiburg	EPI_ISL_852795	Medical center Freiburg	EPI_ISL_852686	Medical center Freiburg
EPI_ISL_852782	Medical center Freiburg	EPI_ISL_852790	Medical center Freiburg	EPI_ISL_852685	Freiburg area
EPI_ISL_852759	Medical center Freiburg	EPI_ISL_852774	Medical center Freiburg	EPI_ISL_852684	Medical center Freiburg
EPI_ISL_852758	Medical center Freiburg	EPI_ISL_852770	Medical center Freiburg	EPI_ISL_852683	Freiburg area
EPI_ISL_852812	Freiburg area	EPI_ISL_852779	Medical center Freiburg	EPI_ISL_852682	Medical center Freiburg
EPI_ISL_852755	Medical center Freiburg	EPI_ISL_852668	Medical center Freiburg	EPI_ISL_852810	Medical center Freiburg
EPI_ISL_852754	Medical center Freiburg	EPI_ISL_852798	Medical center Freiburg	EPI_ISL_852681	Medical center Freiburg
EPI_ISL_852749	Freiburg area	EPI_ISL_852797	Medical center Freiburg	EPI_ISL_852680	Medical center Freiburg
EPI_ISL_852747	Freiburg area	EPI_ISL_852796	Medical center Freiburg	EPI_ISL_852679	Medical center Freiburg
EPI_ISL_852746	Freiburg area	EPI_ISL_852793	Medical center Freiburg	EPI_ISL_852678	Freiburg area
EPI_ISL_852745	Freiburg area	EPI_ISL_852788	Medical center Freiburg	EPI_ISL_852677	Freiburg area
EPI_ISL_852742	Freiburg area	EPI_ISL_852760	Freiburg area	EPI_ISL_852676	Freiburg area
EPI_ISL_852803	Medical center Freiburg	EPI_ISL_852757	Freiburg area	EPI_ISL_852806	Immunosuppressed patient (day 140)
EPI_ISL_852740	Freiburg area	EPI_ISL_852756	Freiburg area	EPI_ISL_852809	Immunosuppressed patient (day 56)
EPI_ISL_852794	Medical center Freiburg	EPI_ISL_852753	Freiburg area	EPI_ISL_852807	Immunosuppressed patient (day 105)
EPI_ISL_852791	Medical center Freiburg	EPI_ISL_852752	Freiburg area	EPI_ISL_852805	Immunosuppressed patient (day 71)
EPI_ISL_852789	Medical center Freiburg	EPI_ISL_852751	Freiburg area	EPI_ISL_852808	Immunosuppressed patient (day 59)
EPI_ISL_852787	Medical center Freiburg	EPI_ISL_852750	Freiburg area	EPI_ISL_852667	Immunosuppressed patient (day 14)
EPI_ISL_852734	Medical center Freiburg	EPI_ISL_852739	Freiburg area	EPI_ISL_852659	Immunosuppressed patient (day 42)
EPI_ISL_852730	Freiburg area	EPI_ISL_852736	Freiburg area	EPI_ISL_852709	Immunosuppressed patient (day 0)
EPI_ISL_852780	Medical center Freiburg	EPI_ISL_852735	Freiburg area	EPI_ISL_852671	Immunosuppressed patient (day 7)
EPI_ISL_852777	Freiburg area	EPI_ISL_852768	Medical center Freiburg		
EPI_ISL_852776	Medical center Freiburg	EPI_ISL_852675	Medical center Freiburg		
EPI_ISL_852728	Medical center Freiburg	EPI_ISL_852674	Medical center Freiburg		
EPI_ISL_852726	Medical center Freiburg	EPI_ISL_852767	Medical center Freiburg		
EPI_ISL_852725	Freiburg area	EPI_ISL_852673	Freiburg area		
EPI_ISL_852724	Freiburg area	EPI_ISL_852672	Medical center Freiburg		
EPI_ISL_852722	Medical center Freiburg	EPI_ISL_852669	Freiburg area		
EPI_ISL_852721	Medical center Freiburg	EPI_ISL_852764	Freiburg area		

Supplementary Table 3: Detailed list of GISAID accession numbers of the 250 randomly selected sequences from Germany between February and April 2020.

We gratefully acknowledge the following Authors from the Originating laboratories responsible for obtaining the specimens, as well as the submitting laboratories where the genome data were generated and shared via GISAID, on which this research is based.

All Submitters of data may be contacted directly via www.gisaid.org

Authors are sorted alphabetically.

Accession ID	Originating Laboratory	Submitting Laboratory	Authors
EPI_ISL_729516, EPI_ISL_729520, EPI_ISL_729524, EPI_ISL_729525, EPI_ISL_729536, EPI_ISL_729541, EPI_ISL_729546, EPI_ISL_729550			
see above	A. Krumbholz,Labor Dr. Krauseund Kollegen MVZ GmbH, Kiel	Charité Universitätsmedizin Berlin, Institut für Virologie	Barbara Mühlemann; Christian Drosten; JuliaSchneider; Jörn Beheim-Schwarzbach; Talitha Veith; Terry Jones; VictorM Corman
EPI_ISL_414521, EPI_ISL_732554, EPI_ISL_732555	Bundeswehr Institute of Microbiology	Bundeswehr Institute of Microbiology	AlexandraRehn; Enrico Georgi; Malena Bestehorn-Willmann;MarkusAntwerpen; Markus H Antwerpen and Roman Wölfel; Mathias CWalter; Mathias Walter; Roman Wölfel; Sabine Zange
EPI_ISL_414508, EPI_ISL_417458, EPI_ISL_417468, EPI_ISL_419552, EPI_ISL_425123, EPI_ISL_425125, EPI_ISL_523934, EPI_ISL_523940, EPI_ISL_523945, EPI_ISL_523949			
see above	Center of Medical Microbiology,Virology, and Hospital Hygiene, University of Duesseldorf	Center of Medical Microbiology,Virology, and Hospital Hygiene, University of Duesseldorf	Alexander Dilthey; Andreas Walker; Björn-ErikJensen; Daniel Strelow; Detlef Kindgen-Milles;Hendrik Streeck; JessicaNicolai; Jörg Timm; Klaus Pfeffer; Malte Kohns Vasconcelos; Marcel Andree; Marek Korencak; Maximilian Damagnez; Ortwin Adams; Sandra Hauka; Tina Senff; Tobias Wienemann; Torsten Feldt; TorstenHouwaart
EPI_ISL_516630, EPI_ISL_516631, EPI_ISL_516632, EPI_ISL_516639, EPI_ISL_516640, EPI_ISL_516644	Charité Universitätsmedizin Berlin, Institut für Virologie/LaborBerlin	Charité Universitätsmedizin Berlin, Institut für Virologie/LaborBerlin	Barbara Mühlemann; Christian Drosten; JuliaSchneider; Jörn Beheim-Schwarzbach; Talitha Veith; Terry Jones; VictorM Corman
EPI_ISL_729477, EPI_ISL_729489, EPI_ISL_729491, EPI_ISL_729514, EPI_ISL_729530, EPI_ISL_753798, EPI_ISL_753811, EPI_ISL_753924, EPI_ISL_753937, EPI_ISL_753942, EPI_ISL_754026, EPI_ISL_754028, EPI_ISL_754034, EPI_ISL_754041, EPI_ISL_754046, EPI_ISL_754050, EPI_ISL_806528, EPI_ISL_806530, EPI_ISL_806532, EPI_ISL_806533, EPI_ISL_806539			
see above	Charité Universitätsmedizin Berlin, Institut für Virologie/LaborBerlin	Charité Universitätsmedizin Berlin, Institut für Virologie	Barbara Mühlemann; Christian Drosten; Cornelia Schlee; Julia Schneider; Jörn Beheim-Schwarzbach; Talitha Veith; Terry Jones; Tomasz Zemojtel; Victor M Corman
EPI_ISL_513299, EPI_ISL_513300	Department of Infection Prevention and Infectious Diseases, University Hospital Regensburg	University Hospital Regensburg	Fritsch, J.; Holzmann,T.; Schneider-Brachert, W.
EPI_ISL_602282, EPI_ISL_602288, EPI_ISL_602293, EPI_ISL_602294	Evangelisches Klinikum Bethel, Institut für Laboratoriumsmedizin, Mikrobiologie und Hygiene	Bielefeld University	Alexander Sczyrba; Christiane Scherer; David Brandt; Jörn Kalinowski;Levin-JoeKlages; Marina Simunovic; Markus Haak; Svenja Vinke; Tobias Busche
EPI_ISL_883163, EPI_ISL_883164, EPI_ISL_883167	Hannover Medical School, Institute of Virology	Hannover Medical School, Institute of Virology	Jasper Götting; Lars Steinbrück
EPI_ISL_602469, EPI_ISL_602470, EPI_ISL_602471, EPI_ISL_602474, EPI_ISL_602475, EPI_ISL_602476, EPI_ISL_602479, EPI_ISL_602482, EPI_ISL_602485, EPI_ISL_602488, EPI_ISL_602498, EPI_ISL_602504, EPI_ISL_602505, EPI_ISL_602506, EPI_ISL_602507, EPI_ISL_602519, EPI_ISL_602524, EPI_ISL_626217			
see above	Institute for Virology,University Hospital Essen	Center of Medical Microbiology,Virology, and Hospital Hygiene, University of Duesseldorf	Alexander Dilthey; Andreas Walker; Daniel Strelow; JessicaNicolai;Jörg Timm; Klaus Pfeffer; Lisanna Hülse; Malte Kohns Vasconcelos; Maximilian Damagnez; Nadine Lübke; Olympia E. Anastasiou; Tobias Wienemann; Torsten Houwaart; Ulf Dittmer
EPI_ISL_525473	Institute of Clinical Microbiology and Hygiene, University Hospital Regensburg	Institute of Clinical Microbiology and Hygiene, University Hospital Regensburg	Hiergeist, A.
EPI_ISL_723077, EPI_ISL_723107, EPI_ISL_723114, EPI_ISL_723115	Institute of Medical Geneticsand AppliedGenomics	Institute of Medical Geneticsand AppliedGenomics	Angel Angelov; Caspar Gross; Daniela Bezdan; Michael Bitzer; Michael Sonnabend; Michaela Pogoda; Nicolas Casadei; Siri Göpel; Stephan Ossowski; Thomas Iftner; TinaGanzenmüller
EPI_ISL_983571, EPI_ISL_983574, EPI_ISL_983578, EPI_ISL_983580, EPI_ISL_983581, EPI_ISL_983582, EPI_ISL_983587			
see above	Institute ofVirology, University Hospital, University of Bonn and German Center for InfectionResearch (DZIF), Bonn-Cologne,Bonn, Germany	Institute ofVirology, University Hospital, University of Bonn and German Center for InfectionResearch (DZIF), Bonn-Cologne,Bonn, Germany	Marek Korencak et al
EPI_ISL_763086	Jena University Hospital, Institute for Infectious Diseases and Infection Control	Institute of infectious medicine & hospital hygiene, CaSe-Group	Brandt; Christian; Marquet; Matthias W.; Mike; Pletz;Riccardo; Spott
EPI_ISL_631300	MVZ DIAMEDISDiagnostische MedizinSennestadt GmbH	Bielefeld University	Alexander Sczyrba; Christiane Scherer; David Brandt; Jörn Kalinowski;Levin-JoeKlages; Marina Simunovic; Markus Haak; Svenja Vinke; Tobias Busche
EPI_ISL_640223, EPI_ISL_640226, EPI_ISL_640228, EPI_ISL_640236, EPI_ISL_640247, EPI_ISL_640252, EPI_ISL_640253, EPI_ISL_640261			
see above	MVZ Laborärzte Singen	MVZ Laborärzte Singen	Folker Wenzel; FrithjofBlessing; Jonas Schmidt; Sandro Berghaus
EPI_ISL_420899, EPI_ISL_420901, EPI_ISL_420902, EPI_ISL_420907, EPI_ISL_420912	Max von Pettenkofer Institute, Virology,National Reference Center for Retroviruses, LMU Munich	Laboratory for Functional Genome Analysis,Dept. Genomics, Gene Center of the LMU Munich	Alexander Graf; Ashok Varadharajan; Helmut Blum; Max Muenchhoff; Oliver Keppler; Stefan Krebs
EPI_ISL_437215, EPI_ISL_437217, EPI_ISL_437221, EPI_ISL_437241, EPI_ISL_437245, EPI_ISL_437248, EPI_ISL_437249, EPI_ISL_437260, EPI_ISL_437266, EPI_ISL_437270, EPI_ISL_437271, EPI_ISL_437272, EPI_ISL_437274, EPI_ISL_437276, EPI_ISL_437278, EPI_ISL_437283, EPI_ISL_437287, EPI_ISL_437290, EPI_ISL_437292, EPI_ISL_437295, EPI_ISL_451944, EPI_ISL_466888, EPI_ISL_466892			
see above	Max von Pettenkofer Institute, Virology,National Reference Center for Retroviruses, LMU München	Laboratory for Functional Genome Analysis,Dept. Genomics, Gene Center of the LMU Munich	Alexander Graf; Helmut Blum; Max Muenchhoff;Oliver Keppler; Stefan Krebs
EPI_ISL_490205, EPI_ISL_490207	München Klinik Schwabing	MGZ Medical Genetics Center	Dieter A. Wolf; Elke Holinski-Feder
EPI_ISL_887331, EPI_ISL_887335, EPI_ISL_887353, EPI_ISL_887354, EPI_ISL_887357, EPI_ISL_887360, EPI_ISL_887369, EPI_ISL_887370, EPI_ISL_887379, EPI_ISL_887386, EPI_ISL_887395, EPI_ISL_887397, EPI_ISL_887400, EPI_ISL_887403, EPI_ISL_887415			
see above	Protzer Lab	Protzer Lab, Gagneur Lab, Robert Koch Institut	Aleksandar Radonic; Alexander Karollus;Andrea Theumer; Dieter Hoffmann; EvaSchulte; Julien Gagneur; Max von Kleist; Oliver Drechsel; Stephan Fuchs; Ulrike Protzer
EPI_ISL_883179, EPI_ISL_883182, EPI_ISL_883184, EPI_ISL_883188	UK Tübingen, Medical Microbiology	Hannover Medical School, Institute of Virology	Jasper Götting; Lars Steinbrück
EPI_ISL_775905, EPI_ISL_775911, EPI_ISL_775914, EPI_ISL_775918, EPI_ISL_775941, EPI_ISL_775944, EPI_ISL_775949, EPI_ISL_775958, EPI_ISL_775967, EPI_ISL_775968, EPI_ISL_775974, EPI_ISL_775977, EPI_ISL_775979, EPI_ISL_775983, EPI_ISL_775996, EPI_ISL_775998, EPI_ISL_776002, EPI_ISL_776017, EPI_ISL_776020, EPI_ISL_776035, EPI_ISL_776038, EPI_ISL_776040, EPI_ISL_776048, EPI_ISL_776061, EPI_ISL_776066, EPI_ISL_776070, EPI_ISL_776076, EPI_ISL_776079, EPI_ISL_776082, EPI_ISL_776087, EPI_ISL_776093, EPI_ISL_776094, EPI_ISL_776095, EPI_ISL_776100, EPI_ISL_776103, EPI_ISL_776105, EPI_ISL_776106, EPI_ISL_776108, EPI_ISL_776122, EPI_ISL_776123, EPI_ISL_776125, EPI_ISL_776127, EPI_ISL_776134, EPI_ISL_776140, EPI_ISL_776157, EPI_ISL_776160, EPI_ISL_776170, EPI_ISL_776172, EPI_ISL_776178, EPI_ISL_776191, EPI_ISL_776192, EPI_ISL_776193, EPI_ISL_776201, EPI_ISL_776214, EPI_ISL_776227, EPI_ISL_776230, EPI_ISL_776231, EPI_ISL_776237, EPI_ISL_776242, EPI_ISL_776246, EPI_ISL_776253, EPI_ISL_776254, EPI_ISL_776257, EPI_ISL_776258, EPI_ISL_776260, EPI_ISL_776261, EPI_ISL_776262, EPI_ISL_776266, EPI_ISL_776270, EPI_ISL_776275, EPI_ISL_776286, EPI_ISL_776292, EPI_ISL_776293, EPI_ISL_776295, EPI_ISL_776312, EPI_ISL_776313, EPI_ISL_776315, EPI_ISL_776317, EPI_ISL_776320, EPI_ISL_776322, EPI_ISL_776326, EPI_ISL_776340, EPI_ISL_776345, EPI_ISL_776355, EPI_ISL_776360, EPI_ISL_776395, EPI_ISL_776397, EPI_ISL_776410, EPI_ISL_776411, EPI_ISL_776424, EPI_ISL_776427, EPI_ISL_776434, EPI_ISL_776454, EPI_ISL_776473, EPI_ISL_776474, EPI_ISL_776478, EPI_ISL_776481, EPI_ISL_776491, EPI_ISL_776493, EPI_ISL_776501, EPI_ISL_776505, EPI_ISL_776507, EPI_ISL_776515, EPI_ISL_776557			
see above	University Medical Center Hamburg Eppendorf	Heinrich Pette Institute, LeibnizInstitute for Experimental Virology	Adam Grundhoff; AlexisRobitaille; Johannes Knobloch; Martin Aepfelbacher; Nicole Fischer; Thomas Günther
