# Supplementary material for: Within-host evolution of SARS-CoV-2 in an immunosuppressed COVID-19 patient as a source of immune escape variants
Source: Nat Commun. 2021 Nov 4;12:6405. doi: 10.1038/s41467-021-26602-3 (PMC8568958; doi:10.1038/s41467-021-26602-3)
Supplement: Supplementary file 3 — Reporting Summary [file 41467_2021_26602_MOESM3_ESM.pdf]

nature research

Corresponding author(s): Georg Kochs

Last updated by author(s): Sep 24, 2021

Reporting Summary

Nature Research wishes to improve the reproducibility of the work that we publish. This form provides structure for consistency and transparency in reporting. For further information on Nature Research policies, see our Editorial Policies and the Editorial Policy Checklist.

Statistics

For all statistical analyses, confirm that the following items are present in the figure legend, table legend, main text, or Methods section.

n/a	Confirmed	
☐	☒	The exact sample size (*n*) for each experimental group/condition, given as a discrete number and unit of measurement
☐	☒	A statement on whether measurements were taken from distinct samples or whether the same sample was measured repeatedly
☐	☒	The statistical test(s) used AND whether they are one- or two-sided<br>*Only common tests should be described solely by name; describe more complex techniques in the Methods section.*
☒	☐	A description of all covariates tested
☒	☐	A description of any assumptions or corrections, such as tests of normality and adjustment for multiple comparisons
☐	☒	A full description of the statistical parameters including central tendency (e.g. means) or other basic estimates (e.g. regression coefficient) AND variation (e.g. standard deviation) or associated estimates of uncertainty (e.g. confidence intervals)
☐	☒	For null hypothesis testing, the test statistic (e.g. *F*, *t*, *r*) with confidence intervals, effect sizes, degrees of freedom and *P* value noted<br>*Give P values as exact values whenever suitable.*
☒	☐	For Bayesian analysis, information on the choice of priors and Markov chain Monte Carlo settings
☒	☐	For hierarchical and complex designs, identification of the appropriate level for tests and full reporting of outcomes
☒	☐	Estimates of effect sizes (e.g. Cohen's *d*, Pearson's *r*), indicating how they were calculated

Our web collection on statistics for biologists contains articles on many of the points above.

Software and code

Policy information about availability of computer code

Data collection	Full-length SARS-CoV-2 genome sequences from Germany were downloaded from GISAID (www.gisaid.org) and randomly down-sampled. The GISAID accession numbers and originating laboratories are acknowledged in the manuscript.<br>All software used to perform data collection are described in the methods section of the manuscript or the supportive information.<br>Multiparametric Flow cytometry data was collected by FACSDiva version 8.0.1 (Becton Dickinson) and CytExpert Software.
Data analysis	Protein structure visualization: UCSF ChimeraX version: 1.1 (2020-09-09); Data analysis, visualization and statistical analysis: Prism8 version 8.4.2 (679) (08.04.20), Image recording and processing: ZEN 2.6 blue edition, Carl Zeiss Microscopy GmbH, Version 2.6.76.00000 and ImageJ software version 1.53c (Rasband, W.S., ImageJ, U. S. National Institutes of Health, Bethesda, Maryland, USA); In silico prediction of antigenic peptides: ANN 4.0 on the Immune Epitope Database website (Nielsen, M., et al. Protein Sci 12, 1007-1017 (2003); Multiparametric Flow cytometry data was analyzed using FlowJo software version 10.6.2 (Treestar, Becton Dickinson).<br>For the phylogenetic analysis, the sequences were first aligned with MAFFT (v7.45) and a tree was constructed with IQ-Tree (v2.1.2);<br>SARS-CoV-2 NGS data was analyzed on the cloud computing bioinformatic platform Galaxy (usegalaxy.eu, covid19.galaxyproject.org/artic/) using the following pipline: fastqs were preprocessed with fastp (v.0.20.1) and mapped using BWA-MEM (v.0.7.17), ARTIC primer sequences were trimmed using ivar trim (v1.9), SNPs and INDELs were called with lofreq (v2.1.5) and annotated with snpeff (v.4.3.1). Consensus sequences were generated with bcftools (v.1.10); Visualization of the variant frequencies and phylogenetic trees were performed with R (v.4.0.2), the R code (release v.1.0) is available on Github (github.com/jonas-fuchs/SARS-CoV-2-analyses).

For manuscripts utilizing custom algorithms or software that are central to the research but not yet described in published literature, software must be made available to editors and reviewers. We strongly encourage code deposition in a community repository (e.g. GitHub). See the Nature Research guidelines for submitting code & software for further information.

Data

Policy information about availability of data

All manuscripts must include a data availability statement. This statement should provide the following information, where applicable:

- Accession codes, unique identifiers, or web links for publicly available datasets
- A list of figures that have associated raw data
- A description of any restrictions on data availability

All necessary data and informations are given in the manuscript. SARS-CoV-2 consensus sequences have been deposited to GISAID database (www.gisaid.org). Accession numbers are given in the manuscript (Supplementary table 2). Raw sequencing data have been submitted to the European Nucleotide Archive (https://www.ebi.ac.uk/ena/browser) under the study accession number: ERP132087.
EM structure of the closed conformation of D614G SARS-CoV-2 spike protein was loaded from the protein data bank (10.2210/pdb7BNM/pdb) and visualized with UCSF ChimeraX version: 1.1 (2020-09-09).
In silico peptide binding was analyzed with ANN 4.0 on the Immune Epitope Database website (https://www.iedb.org/).
For requests, please contact G. Kochs, georg.kochs@uniklinik-freiburg.de, and M. Panning, marcus.panning@uniklinik-freiburg.de. Requests will be processed within a week.
Restrictions: Further additional information about the patient will not be shared due to protection of individuals‘ privacy.

Field-specific reporting

Please select the one below that is the best fit for your research. If you are not sure, read the appropriate sections before making your selection.

☒ Life sciences ☐ Behavioural & social sciences ☐ Ecological, evolutionary & environmental sciences

For a reference copy of the document with all sections, see nature.com/documents/nr-reporting-summary-flat.pdf

Life sciences study design

All studies must disclose on these points even when the disclosure is negative.

Sample size	-Sample sizes were not predictable in advance because of the limited number of immunosuppressed COVID-19 patients and convalescent COVID-19 patients that agreed to provide sera for this study.<br>- A single 58-years old male, kidney transplant patient was studied from March, 2020, to September 2020, at the University Hospital Freiburg.<br>- Immunocompetent patients were recruited and patient material was banked at the University Hospital Freiburg; inclusion criteria were: COVID-19 convalescent individuals following a mild to severe course of SARS-CoV-2 infection, SARS-CoV-2 infection was confirmed by positive PCR testing from oropharyngeal swab and/or SARS-CoV-2 spike IgG positive antibody testing.<br>- To show the immune escape of the patient's late isolate (d105) we used sera from 3 to 11 individuals for the different serological assays. The samples sizes were determined by the limited accessibility of sera from convalescent or vaccinated individuals early in the pandemic.<br>- For infection experiments with laboratory mice, 7-8 animals were used per group as recommended by the animal protection proposal approved by the local animal welfare committee. In the subsequent challenging experiments the numbers of animals were limited due to the small number of animals that survived the initial infection.
Data exclusions	No data were excluded.
Replication	Data were reproduced as biological triplicates with technical duplicates in independent experiments.
Randomization	- Accessible human antisera were choosen for the different serological assays according to the severity of COVID-19 or the history of vaccination to achive a diverse set of antisera.<br>To analyze A*02/S133, A*02/S142, A*02/S244, A*03/S142 and A*03/S378-specific T cell responses participants needed to be allocated into experimental groups based on their HLA types (A*02 and A*03, respectively). In the present study, the covariates age and gender are not relevant for the experiments, as they have no direct influence on the experimental design to detect the presence of epitope-specific T cells.<br>- mice were allocated randomly concerning age and sex into the experimental groups.
Blinding	Only objective parameters were included in the study design. Blinding was not applied. Non-objective parameters were not included in the study design. Due to standardized analyses of the flow cytometric data set, biased analysis can be excluded.

Reporting for specific materials, systems and methods

We require information from authors about some types of materials, experimental systems and methods used in many studies. Here, indicate whether each material, system or method listed is relevant to your study. If you are not sure if a list item applies to your research, read the appropriate section before selecting a response.

Materials & experimental systems

n/a	Involved in the study	
	x	Antibodies
	x	Eukaryotic cell lines
x		Palaeontology and archaeology
	x	Animals and other organisms
	x	Human research participants
	x	Clinical data
x		Dual use research of concern

Methods

n/a	Involved in the study	
x		ChIP-seq
	x	Flow cytometry
x		MRI-based neuroimaging

Antibodies

Antibodies used

SARS-CoV-2 N-specific, SARS-CoV-2 spike protein (RBD)-specific (200-401-A50, 600-401-MS8, Rockland), actin-specific (A5060, Sigma) rabbit antisera,
AF568-labeled goat-anti-rabbit (Invitrogen, #A11011, 1:400) secondary antibody.
anti-IFNɣ Ab, BD , Cat No 340449, clone 25723.11 , Lot No 9262323
anti-CD8 Ab, BD , Cat No 564804 , clone RPA-T8 , Lot No 9196503

Validation

All antibodies were obtained from commercial vendors and specificity characteristics were based on descriptions and information provided in corresponding data sheets available and provided by the manufacturers.
For the SARS-CoV-2 N and S-specific antibodies: Bouhaddou et al., Cell. 2020 Aug 6;182(3):685-712.e19. doi: 10.1016/j.cell.2020.06.034. Epub 2020 Jun 28.
For validation of the antibodies used in the FACS analysis, we performed standardized analysis in different cohorts, antibody titration on PBMCs including unstained controls, comparisons of different antibody clones and conjugates:
anti-CD8, clone RPA-T8: antibody titration on PBMCs; control clone GHI/75; using B cells as negative control
anti-IFNɣ, clone 25723.11: antibody titration on PBMCs; control clone 4S.B3; validated with respect to differential expression of activated and non-activated T cell subpopulations
Viability Dye was titrated on PBMCs; validated with respect to differential staining of live and dead cell populations

Eukaryotic cell lines

Policy information about cell lines

Cell line source(s)

VeroE6 cells (ATCC CRL-1586), BHK-21 cells (ATCC CCL-10), Calu-3 cells (ATCC-HTB-55) kindly provided by Markus Hoffmann, Cell. 2020 Apr 16;181(2):271-280.e8. doi: 10.1016/j.cell.2020.02.052. Epub 2020 Mar 5)

Authentication

None of the cell lines were authenticated.

Mycoplasma contamination

all cell lines were tested monthly negative for mycoplasma

Commonly misidentified lines
(See ICLAC register)

no commonly misidentified cell lines were used in the study

Animals and other organisms

Policy information about studies involving animals; ARRIVE guidelines recommended for reporting animal research

Laboratory animals

Transgenic (K18-hACE2)2Prlmn mice (Winkler et al., Nat Immunol. 2020 Nov;21(11):1327-1335. doi: 10.1038/s41590-020-0778-2. Epub 2020 Aug 24) were purchased from The Jackson Laboratory and bred locally. Hemizygous 8-12-week-old animals of both sexes were used.
Mice were housed at 14-hour light/10-hour dark cycles and temperatures of ~18-23°C with 40-60% humidity.

Wild animals

no wild animals were used in the study

Field-collected samples

no field collected samples were used in the study

Ethics oversight

All animal studies were performed in accordance with the guidelines of the Federation for Laboratory Animal Science Associations and the National Animal Welfare Body. All experiments were in compliance with the German animal protection law and approved by the animal welfare committee of the Regierungspraesidium Freiburg (permit G-20/91).

Note that full information on the approval of the study protocol must also be provided in the manuscript.

Human research participants

Policy information about studies involving human research participants

Population characteristics	A single, 58-year-old male patient with a history of autosomal dominant polycystic kidney disease (ADPKD) was hospitalized at the University Medical Center, Freiburg, Germany.<br>Population characteristics of the blood donors analyzed in figure 5 and supplementary figure 3: Two patients with severe COVID-19/ARDS, 50 and 61 years, male, used in panel 5a as positive control and in panel 5b, respectively; three patients with moderate COVID-19/O2 demand, 83-90 years, two males, one female, used in panel 5b, 5c, and 5d; twelve patients with mild COVID-19, 25-65 years, used in oanel 5b and suppl. fig. 3. Vaccine sera were from three healthy, female individuals, 31-46 years, used in panel 5b. Serum 11 in sup. fig.3 was from a male, in his fifties, after mild COVID-19 and a following Astra-Zeneca vaccination.
Recruitment	Diagnostic specimens of the immunosuppressed patient were analyzed in this study due to his long-term SARS-CoV-2 infection.<br>Patients and vaccinees were recruited at the University Hospital Freiburg (in- and outpatient section); self-selection bias or other biases can be excluded since several people were included in the recruitment.<br>For T-cell analysis samples were banked and retrospectively selected according to the following inclusion criteria: HLA-A*02:01, HLA-A*03:01
Ethics oversight	The project has been approved by the University Medical Center, Freiburg, ethical committee. Written informed consent was obtained from all participants and the study was conducted according to federal guidelines, local ethics committee regulations (Albert-Ludwigs-Universität, Freiburg, Germany: No. F-2020-09-03-160428 and no. 322/20).<br>All routine virological laboratory testing of patient specimens was performed in the Diagnostic Department of the Institute of Virology, University Medical Center, Freiburg (Local ethics committee no. 1001913).

Note that full information on the approval of the study protocol must also be provided in the manuscript.

Clinical data

Policy information about clinical studies

All manuscripts should comply with the ICMJEguidelines for publication of clinical research and a completedCONSORT checklist must be included with all submissions.

Clinical trial registration	n.a.
Study protocol	n.a.
Data collection	Diagnostic specimens were collected between 20.03.20 to 28.09.20 during the standard care of the hospitalized patient.
Outcomes	n.a.

Flow Cytometry

Plots

Confirm that:

- [x] The axis labels state the marker and fluorochrome used (e.g. CD4-FITC).
- [x] The axis scales are clearly visible. Include numbers along axes only for bottom left plot of group (a 'group' is an analysis of identical markers).
- [x] All plots are contour plots with outliers or pseudocolor plots.
- [x] A numerical value for number of cells or percentage (with statistics) is provided.

Methodology

Sample preparation	Cryopreserved isolated human PBMCs were thawed and prepared for flow cytometry or in vitro expansion described in the methods section.
Instrument	FACSCanto II (BD, Germany) or CytoFLEX (Beckman Coulter)
Software	FlowJo_v10.6.2 (Treestar)
Cell population abundance	Abundance of SARS-CoV-2-specific CD8+ T cells are low (<10^-4 %; Schulien et al., Nat Med, 2021)

Gating strategy	Lymphocytes gated on FSC-A and SSC-A, Doublet exclusion on FSC-A and FSC-H and FSC-A and FSC-W, Exclusion of dead cells, gating on CD8. Gating of SARS-CoV-2-specific CD8+ T cells via IFNγ described in methods part.

☒ Tick this box to confirm that a figure exemplifying the gating strategy is provided in the Supplementary Information.
